# The Gengnianchun recipe attenuates insulin resistance-induced diminished ovarian reserve through inhibiting the senescence of granulosa cells

**DOI:** 10.3389/fendo.2023.1133280

**Published:** 2023-03-01

**Authors:** Hongna Gao, Lingyun Gao, Yanqiu Rao, Laidi Qian, Mingqing Li, Wenjun Wang

**Affiliations:** ^1^ Department of Integrated Traditional & Western Medicine, Obstetrics and Gynecology Hospital of Fudan University, Shanghai, China; ^2^ Department of Integrated Traditional & Western Medicine, Shanghai Key Laboratory of Female Reproductive Endocrine Related Diseases, Shanghai, China

**Keywords:** insulin resistance, diminished ovarian reserve, granulosa cells, cell senescence, traditional Chinese medicine

## Abstract

**Introduction:**

Insulin resistance (IR) is found in patients with polycystic ovary syndrome (PCOS), but the effects and mechanisms of IR on diminished ovarian reserve (DOR) remain unclear. This study set out to investigate the effects of IR on ovarian reserve; to explore the effects of high concentrations of insulin on the function of ovarian cells *in vitro*; and to validate the hypothesis that the Gengnianchun recipe (GNC) helps to attenuate DOR caused by IR through reducing the senescence of granulosa cells.

**Methods:**

Estrus cycle, follicle count, and sex hormone levels were detected to evaluate ovarian function in mice with IR caused by feeding a high-fat diet (HFD). In addition, KGN cells (human granulosa cell line) were treated with high concentrations of insulin. The staining for senescence-associatedβ-galactosidase (SA-β-gal), cell cycle, and expression levels of mRNA and gene proteins related to cell aging were detected in KGN cells treated with high concentrations of insulin. Mice treated with an HFD were fed metformin, GNC, or saline solution for 6 weeks by oral gavage. HOMA-IR, the area under the curve (AUC) of the oral glucose tolerance test (OGTT), levels of fasting blood glucose (FBG), and fasting serum insulin (FINS) were examined to confirm the IR status. Then estrus cycle, follicle count, and sex hormone levels were detected to evaluate ovarian function. Expression levels of mRNA and gene proteins related to cell aging were detected in the ovarian tissue of mice in each group.

**Results:**

The results demonstrated that IR reduced murine ovarian reserves, and high doses of insulin caused granulosa cells to senesce. There was a considerable improvement in HFD-induced IR status in the metformin (Met) and GNC treatment groups. In addition, the expression levels of aging-associated biomarkers were much lower in GNC mice than Met mice; and both the latter groups had considerably lower levels than the HFD group. Moreover, higher follicle counts in different stages and shorter diestrus in the Met or GNC groups compared to the HFD group indicated that ovarian aging could be largely reversed.

**Discussion:**

This work showed that: IR impaired ovarian reserve; high concentrations of insulin induced granulosa cell aging; and GNC attenuated ovarian function through inhibiting IR-induced cell aging.

## 1 Introduction

Diminished ovarian reserve (DOR), which is defined as a decrease in the number or quality of ovarian follicles, plays a major role in female infertility and increased miscarriage rates (1, 2). Unfortunately, there is a significant upward trend in DOR prevalence. The prevalence of DOR in infertile patients attending *in vitro* fertilization centers in the United States increased from 19% to 26%, while diagnoses among patients <40 y have increased by 42% from 2004 to 2011 (3). DOR is characterized by a lower number of antral follicles, an elevated level of serum follicle-stimulating hormone (FSH), and decreased levels of serum estradiol (E2) and anti-Müllerian hormone (AMH).

DOR can be caused by genetic factors, environmental pollutants, infections, and chronic stress. However, in our clinical practice, we find that DOR insidiously increases with the development of PCOS and insulin resistance (IR). IR and hyperinsulinemia are closely associated and occur concurrently (4), which supports findings that compensatory hyperinsulinemia can be an adverse side effect of insulin resistance. IR and hyperinsulinemia are associated with polycystic ovary syndrome (PCOS) (5). Earlier studies also support positive associations between DOR and HOMA-IR in patients and mice (6, 7). However, the adverse effects of IR on DOR and the detailed pathogenic mechanisms of IR remain elusive.

Cellular senescence, one of the hallmarks of aging, leads to age-related disease and dysfunction (8). Senescent cells, triggered by a variety of stressors including telomere dysfunction and genotoxic and oxidative stress, are characterized by a state of irreversible cell-cycle arrest, secretion of senescence-associated secretory phenotypes (SASPs), and increased senescence‐associated β‐galactosidase (SA-β-Gal) activity (9). Moreover, hyperinsulinemia leads to cell-cycle-induced senescence, which has been demonstrated on neurons in an Alzheimer’s disease mouse model (10). In *in vitro* studies and in humans, chronic hyperinsulinemia results in cell cycle exit and a premature senescence of adipocytes, and this trajectory has been reversed by the administration of metformin (11). Several human and mouse studies emphasize the presence of senescence in human hepatocyte cells and the involvement of senescent cells in the development and progression of non-alcoholic fatty liver disease (12, 13). The presence of cell cycle arrest in granulosa cells has been confirmed in patients with premature ovarian insufficiency *in vitro* and *in vivo*, although cell senescence in that case was caused by reactive oxygen species (14).

Gengnianchun (GNC), a traditional Chinese medicine (TCM) formula, is composed of *Radix rehmanniae*, *Rhizoma coptidis*, *Radix paeoniae alba*, *Rhizoma anemarrhenae*, *Cistanche salsa, Radix morindae officinalis, Poria, Epimedium brevicornum, Cortex phellodendri amurensis, Fructus lycii, Semen cuscutae*, and *Carapax et plastrum testudinis* (15). According to TCM theory, GNC has a kidney/liver tonifying effect that is used to alleviate declining functions related to aging. To date, GNC has been shown to have beneficial effects on aging-related conditions such as menopause and Alzheimer’s disease (16, 17). GNC has also been shown to improve learning and memory, delay skin aging, and enhance resistance to oxidative stress (18, 19). Considering these findings, GNC might be able to delay the process of aging. The GNC dose selection for treatment in this study was based on our previous study, which indicated that GNC can significantly preserved the ovarian reserve of rats (20). GNC was also shown to modulate the hypothalamus-pituitary-ovary axis, and increase estradiol receptor (ER) levels in the pituitary gland and ovaries (21), but the specific mechanism remains to be elucidated.

In sum, IR reduces ovarian reserve by inducing granulosa cell senescence and GNC seems to be able to protect ovarian function. To test the hypothesis, an IR mouse model was established and the ovarian reserve was evaluated. The underlying mechanism was verified by detecting senescence-associated changes in KGN cells after treatment with high concentrations of insulin. Ovarian reserve in the IR mouse model was assessed after administering GNC by oral gavage.

## 2 Materials and methods

### 2.1 Drugs and reagents

The following drugs and reagents were used in the study: DMEM/F12 media without phenol red (Gibco), fetal bovine serum (FBS, Sciencell), RNA isolation kit, Color SYBR Green qPCR Master Mix and 4× reverse transcription Mix (EZBioscience, A0012), enzyme linked immunosorbent assay (ELISA) kits (E2, Labor Diagnostika Nord, FR E-2000; FSH, Immunoway, KE1425; LH, Immunoway, KE1475; AMH, Jingmei, JM11692M1; insulin, Alpco, 80-INSMSU-E01, E10), Normal rabbit IgG (CST, 2729), Normal mouse IgG (CST, 7076), p53 antibody (Abcam ab131442), p16 antibody (Cell Signaling Technology Cat, sc1661), *p21* antibody (Cell Signaling Technology, sc-6246), AGER antibody (Abcam, ab3611), Senescenceβ-Galactosidase Staining Kit (Beyotime, C0602), Cell Cycle Staining Kit (Multi Sciences, CCS021), insulin (Absin, abs42019847), metformin (Topscience, T8526), high-fat diet (Jiangsu Xietong Pharmaceutical Bio-engineering Co., Ltd., D12451).

### 2.2 Animals

Four-week-old C57BL/6 female mice were purchased from Shanghai Slac Laboratory Animal Ltd. The mice were housed in the SPF facility at a constant temperature (25°C) and humidity (55%) with a 12-h light/dark cycle. After adaptive feeding for 1 week with water and normal feed, all mice were randomly divided into four groups: control group (Ctrl), model group (HFD), metformin group (Met), and GNC group (GNC). Mice in groups HFD, Met, and GNC were fed the high fat diet for 9 weeks according to previous reports and changes in estrus cycle were monitored (22). After 3 weeks, mice in Ctrl and HFD were given normal saline by gavage for 6 weeks, while mice in Met and GNC were given metformin (200 mg/kg·d body weight) and GNC decoction (2.77 g/kg body weight), respectively by gavage for 6 weeks. Metformin was dissolved in sterile water. The mouse dose of GNC was converted from the rat dose according to our previous study and the GNC decoction was dissolved into hot water (20). Oral gavage volumes were adjusted according to body weight, which was measured every 7 d (23), while mice in Ctrl were administered the same volume of solvent solution. Experiments were conducted in accordance with Medical Laboratory Criteria. Animal studies were reviewed and approved by the Animal Experimental Ethical Committee of Fudan University.

### 2.3 Gengnianchun formula

The GNC formula, containing 12 crude herbs, is a conventional medicine for clinical therapy. The GNC formula is composed of *Radix rehmanniae, Rhizoma coptidis, Radix paeoniae alba, Rhizoma anemarrhenae, Cistanche salsa, Radix morindae officinalis, Poria, Epimedium brevicornum, Cortex phellodendri amurensis, Fructus lycii, Semen cuscutae, and Carapax et plastrum testudinis*, which were purchased from Tianjiang Pharmaceutical (Jiangyin, China).

### 2.4 Oral glucose tolerance test and HOMA-IR

The oral glucose tolerance test (OGTT) was performed at 8–10 weeks of age. Overnight (12–14 h) fasted mice with free access to water were orally dosed with 20% dextrose anhydrous. Following glucose solution administration, blood samples were collected from the tail vein and glucose levels were measured at 0, 30, 60, 90, and 120 min. IR was estimated using the homeostasis model assessment for IR (HOMA-IR: fasting serum insulin (FINS) [mU/ml] × fasting blood glucose(FBG) [mmol/l]/22.5).

### 2.5 Estrous cycle examination

Estrus cycles were assessed daily with a vaginal smear using normal saline. Approximately 10 ul of saline solution was drawn into the pipette, which was gently inserted into the vaginal canal. A light microscope was used to observe the vaginal fluid after it was stained with hematoxylin and eosin (H&E). The estrous cycle was classified into four stages: proestrus, estrus, metestrus, and diestrus based on the type of major cells in the vaginal smear. Proestrus is confirmed by predominantly nucleated epithelial cells; estrus is characterized by anucleated keratinized epithelial cells; metestrus shows a combination of anucleated keratinized epithelial cells and neutrophils; and diestrus is characterized by a higher number of neutrophils.

### 2.6 Ovary serial sectioning and follicle counting

Ovarian histological analyses and follicle counts were performed on paraffin embedded sections stained with H&E taken from the largest cross-section of each ovary. Each ovary was serially sectioned using a microtome at 5 um. To quantify the total number of follicles in each ovary, the average of five different sections of each ovary was counted throughout the entire ovary. According to the modified Oktay system, follicles were classified into four stages (24). Counting was repeated three times by different researchers, with each replicate containing 14 mice. After summing the number of follicles obtained by different researchers, an average was then obtained.

### 2.7 Determination of E2, FSH, LH, AMH, and insulin concentrations

Mice were sacrificed after a 12–14-h fast at 13 weeks of age. After clotting at room temperature (RT) for 1 h, blood samples were centrifuged at 2000 g for 15 min. Serum samples were stored at 80°C until analysis was performed. Enzyme-linked immunosorbent assay (ELISA) were used to measure serum hormones following the manufacturer’s instructions. The levels of LH, FSH, AMH, estradiol (E2), and insulin were evaluated using commercial ELISA kits. There were 14 samples in each group and each experiment was performed in triplicate. Diluted samples and serially diluted standard reagents were prepared according to the manufacturer’s instructions. In 96-well plates, standard reagents and samples were added, and then enzyme conjugate was added to each well. The plate was incubated at RT and washed three times with wash buffer. Substrate solution was placed in each well of the plate and the reaction was incubated in the dark. Stop solution was used to stop the reaction. The absorbance of each well was read at 450 nm (Biotek Multisken MK3). Standard curves were used to calculate concentrations.

### 2.8 Immunohistochemistry

The tissue array sections were deparaffinized and rehydrated in a series of ethanol gradient, and the endogenous peroxidase activity was quenched by immersing in methanol containing 0.3% hydrogen peroxide. After heating for 30 min at 100°C in saline sodium citrate for antigen retrieval, the sections were incubated overnight at 4°C with primary antibody after blocking the slices using goat serum. The tissue sections were washed three times. They were then incubated with secondary antibodies against IgG at a 1:100 dilution and then stained with DAPI. A microscope (Olympus BX53; Olympus, Tokyo, Japan) and digital camera (Olympus DP73; Olympus) were used for image collection. To estimate the density of each marker from each mouse, five slides were used, and five random images of each slide were taken to calculate the mean density value with Image Pro-Plus 6.0. Quantification of immunostaining was based on both the percentage of positive cells and the intensity of staining (25). Independently, two senior pathologists blinded to the samples evaluated IHC staining results.

### 2.9 KGN cell culture and treatment

KGN cells (human granulosa-like tumor cell line) were purchased from Guangzhou Saiku Biotechnology Co., Ltd and identified using the STR method. DMEM/F12 media without phenol red containing 10% FBS was used to culture KGN cells. Cells were digested and plated on six-well plates for insulin treatments. When the cells had adhered and reached a suitable density, insulin was added after replacing the culture media with DMEM/F12 and starving the cells for 12 h. The concentration of insulin in this study was chosen according to previous studies (26, 27).

### 2.10 Cell cycle analysis using flow cytometry

For cell cycle distribution, cells were cultured with 0.5 or 1 ug/ml insulin for 72 h. The cells were collected, washed with cold PBS once, and stained with 500 μL DNA Staining Solution and 5 μL Permeabilization Solution in the dark at 37°C for 30 min. For each experiment, 2 × 10^5^ cells were recorded. A flow cytometer (Beckman Coulter) was used to analyze cell cycles.

### 2.11 Senescence-associated b-galactosidase staining

b-Galactosidase staining was performed with a senescence-associated b-Galactosidase Staining Kit (Beyotime, China). PBS was used to wash cells three times and stationary liquid was used to fix them for 15 min at RT. Next, the cells were incubated overnight at 37°C without carbon dioxide in darkness with the working solution containing 5% 5-bromo-4-chloro-3-indolyl-b-d-galactopyrano-side (X-gal). Cells were photographed using a light microscope.

### 2.12 Cellular RNA extraction and real-time PCR

Cells were washed in PBS after treatment with insulin for 72 h. NanoDrop (Thermo Scientific) was used to measure the concentration of RNA extracted with an RNA isolation kit. RNA was reverse transcribed into cDNA under the following conditions: one cycle at 42 °C for 15 min and one cycle at 95 °C for 30 s. RT-PCR reactions were performed under the following conditions: one cycle at 50 °C for 2 min, one cycle at 95 °C for 5 min, followed by 40 cycles at 95 °C for 10 s, and 60 °C for 30 s. Samples were normalized to beta-2 microglobulin (B2M), and the comparative CT (threshold cycle) method used to calculate gene expression levels. The primer sequences are listed in **Table 1**.

**Table 1:** Primer sequences of target genes.

Species	Gene	Direction	Sequence
Human	*B2m*	Forward	GAGGCTATCCAGCGTACTCCA
		Reverse	CGGCAGGCATACTCATCTTTT
Human	*P16*	Forward	GGGTTTTCGTGGTTCACATCC
		Reverse	CTAGACGCTGGCTCCTCAGTA
Human	*P21*	Forward	CGATGGAACTTCGACTTTGTCA
		Reverse	GCACAAGGGTACAAGACAGTG
Human	*P53*	Forward	ACAGCTTTGAGGTGCGTGTTT
		Reverse	CCCTTTCTTGCGGAGATTCTCT
Human	*AGER*	Forward	TTTGAGTCCATCACTAACGTCA
		Reverse	GGTAGATGGCATCAATGAATCG
Human	*INHA*	Forward	GACTTTGCCACTGAGTTGATTT
		Reverse	CGATCAGCATTTCCAATATGCA
Human	*IL6*	Forward	ACTCACCTCTTCAGAACGAATTG
		Reverse	CCATCTTTGGAAGGTTCAGGTTG
Human	*IL8*	Forward	ACTGAGAGTGATTGAGAGTGGAC
		Reverse	AACCCTCTGCACCCAGTTTTC
Human	*TNF*	Forward	GAGGCCAAGCCCTGGTATG
		Reverse	CGGGCCGATTGATCTCAGC
Human	*GM-CSF*	Forward	AGGAGGGAGATCCGGTGTC
		Reverse	TTGCGAGACGTTAATCCTGAC
Mouse	*B2m*	Forward	TTCTGGTGCTTGTCTCACTGA
		Reverse	CAGTATGTTCGGCTTCCCATTC
Mouse	*P21*	Forward	CCATGAGCGCATCGCAATC
		Reverse	CCATGAGCGCATCGCAATC
Mouse	*P53*	Forward	CTCTCCCCCGCAAAAGAAAAA
		Reverse	CGGAACATCTCGAAGCGTTTA
Mouse	*P16*	Forward	CGCAGGTTCTTGGTCACTGT
		Reverse	TGTTCACGAAAGCCAGAGCG
Mouse	*AGER*	Forward	CTTGCTCTATGGGGAGCTGTA
		Reverse	GGAGGATTTGAGCCACGCT

### 2.13 Western blot analysis

In six-well plates, cells were washed twice in PBS, lysed on ice for 10 min with 120 ul RIPA buffer containing PMSF and protease inhibitor cocktail and then centrifuged at 4 °C and 12 000 g for 20 min. The protein concentration was determined by a BCA kit (Beyotime, P0012). A mixture of the protein solution and SDS-PAGE loading buffer was boiled at 97°C for 10 min. Protein samples (20 µg) were transferred to polyvinylidene fluoride membranes after being loaded onto the gel. After blocking with skim milk at RT for 1 h, the membranes were incubated overnight with primary antibody solutions at 4°C. The membranes were incubated with secondary antibody solutions at RT for 1 h followed by three washes with TBST. Then the membranes were washed three times with TBST for 10 min each time. An ImageQuant LAS 4000mini system was used to detect the bands with an enhanced chemiluminescent substrate kit. DAPDH was used as a housekeeping gene, relative quantitative protein expression was assessed using ImageJ.

### 2.14 RNA sequencing and DEGs analysis

RNA-seq was performed to compare the global gene expression between control KGN (n = 3), 0.5 ug/ml insulin-treated KGN (n = 3), and 1 ug/ml insulin-treated KGN (n = 3). Principal component analysis (PCA) was then conducted. Volcano plots of differentially expressed mRNAs (DEMs) were generated. The q-value <0.05 and |fold change (FC)| >2 were set as the standard for selecting differently expressed genes (DEGs). DEMs with log2 (fold change) >0.58 were labeled in red (P < 0.05); DEMs with log2 (fold change) <-0.58 were marked in green (P < 0.05). GO and KEGG analysis of DEGs were performed.

### 2.15 Statistical analysis

Concentrations are expressed as mean ± SD. SPSS 22.0 software was used for the statistical analyses. Each experiment included at least three independent samples and was repeated at least three times. The difference between two groups with equal variance was compared using t-tests. One-way ANOVA was used to compare differences between more than two groups. P<0.05 was taken to indicate a significant difference. Symbols for statistical significance levels: **: P < 0.01; ***: P < 0.001; ****: P < 0.0001.

## 3 Results

### 3.1 The insulin-resistant mouse model was successfully established

**Figure 1:**
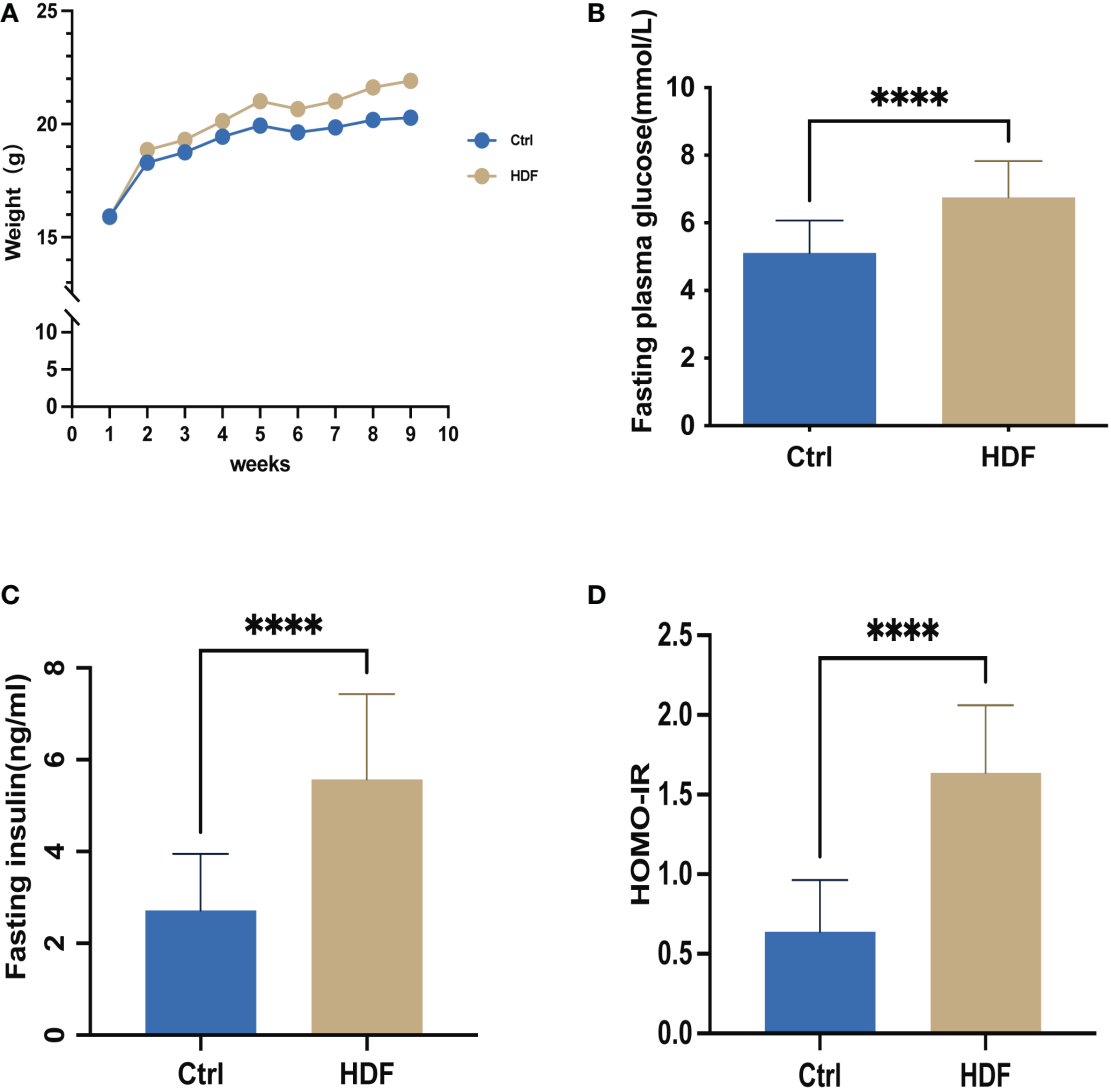
The insulin-resistant mouse model was successfully established. Control group: C57BL/6 mice were fed with a standard diet for 9 weeks; HDF group: C57BL/6 mice were fed with a high fat diet for 9 weeks, respectively. To validate that the insulin-resistant mouse model was successfully established, weight, fasting plasma glucose, and fasting insulin levels in the mice were assessed. **(A)** Weight **(B)** Fasting plasma glucose after 12  h of fasting was determined at the same time point each week, for 8 weeks. n=14 in each group; t-test. **(C)** Fasting insulin concentration was determined by ELISA after 12  h of fasting, n=14 with one repeat in each assay; t-test. **(D)** The HOMA-IR insulin resistance index was calculated, n=14 with one repeat in each assay; t-test. ****:P<0.0001 compared to group “Ctrl”.

To verify the status of IR, we measured the serum insulin and fasting blood glucose levels of each group of mice. As **Figure 1A** shows, the body weight of the HFD-fed mice (HFD) was much higher than for normal diet fed mice (Ctrl). Basal glucose and insulin concentrations were significantly increased in HFD compared with Ctrl following 12–14 h of fasting (**Figures 1B, C**). We also found that HFD had a higher level of significance (P = 0.0001) in HOMA-IR (**Figure 1D**) compared with Ctrl.

### 3.2 Insulin resistance decreased the ovarian reserve

**Figure 2:**
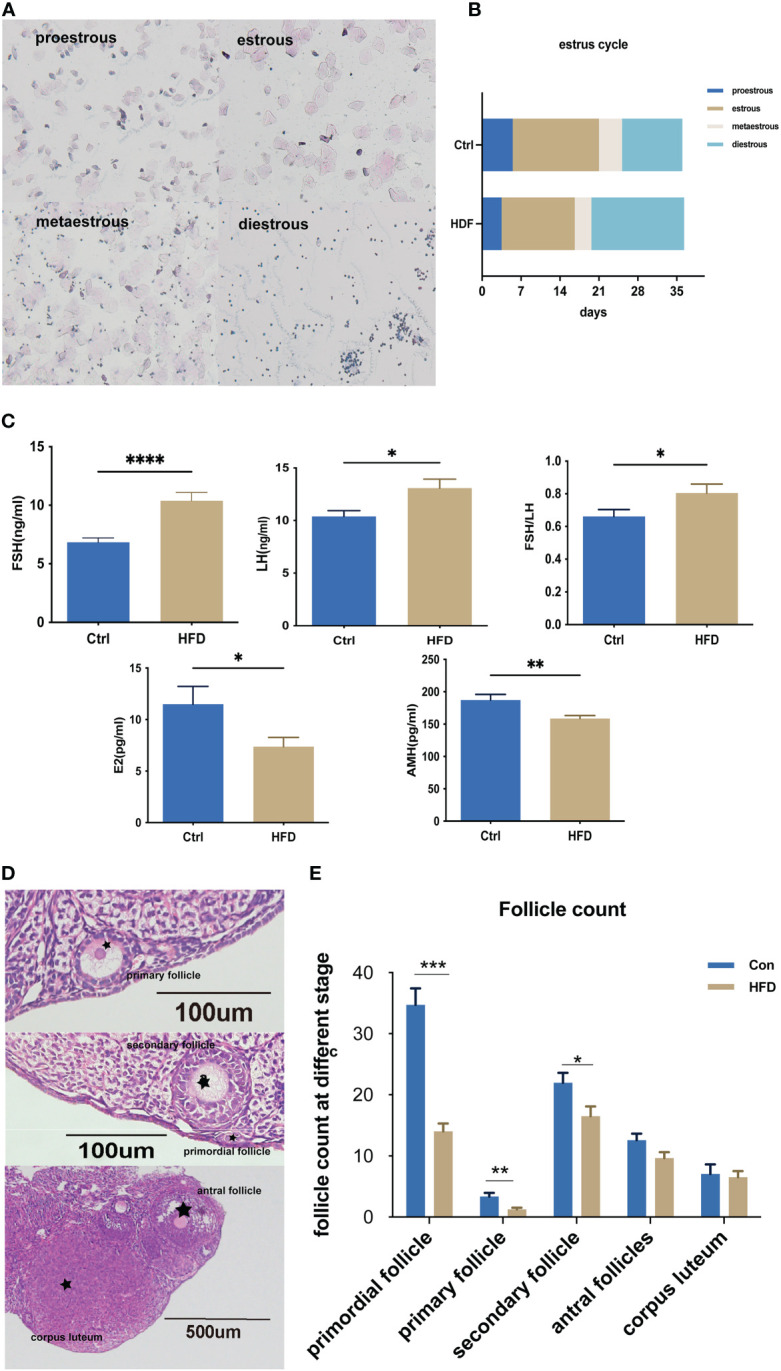
HFD induced-insulin resistance decreased the ovarian reserve. **(A)** Different stages of the estrous cycle were assessed by collecting vaginal smears at the same time point every day. **(B)** Estrus cycles were assessed every day for the last 5 weeks, n=14 with one repeat in each assay. **(C)** Serum FSH, LH, E2, and AMH were determined by ELISA; FSH/LH was calculated, n=14 with one repeat in each assay; t-test. **(D)** Different stages of follicles: primordial follicles; primary follicles, secondary follicles, antral follicles, and corpora lutea. **(E)** Detailed counting of follicles at different stages, n=14 with one repeat in each assay; t-test. *: P<0.05, **: P<0.01, ***: P<0.001, ****: P<0.0001 compared to group “Ctrl”.

We first assessed the estrus cycle using vaginal smears. As shown in **Figure 2A**, the estrus cycle was classified into four stages proestrus, estrus, metestrus, and diestrus. Compared with Ctrl, the percent of diestrus in HFD was much higher (**Figure 2B**). **Figure 2C** shows that the levels of FSH and FSH/LH ratio in HFD were significantly higher than those in Ctrl, while the E2 and AMH concentrations were lower in HFD. Furthermore, we tested the ovarian reserve of those mice by measuring the number of ovarian follicles. **Figure 2D** shows the different stages of follicle growth, including primordial follicle (single oocytes or multi-oocytes surrounded by a thin layer of flattened granulosa cells), primary follicle (oocyte and a layer cubic granulosa cells), secondary follicles (the formation of more than two layers of granulosa cells in the follicles), antral follicles (a fluid‐filled cavity is formed inside each follicle), and corpora lutea (expulsion of a mature oocyte). As shown in **Figure 2E**, the numbers of primordial follicles (P < 0.001), primary follicles (P < 0.01) and secondary follicles (P < 0.05) in HFD were significantly lower than those of the control group.

### 3.3 Long-time exposure of KGN cells to high dose insulin induced granulosa cell senescence

**Figure 3:**
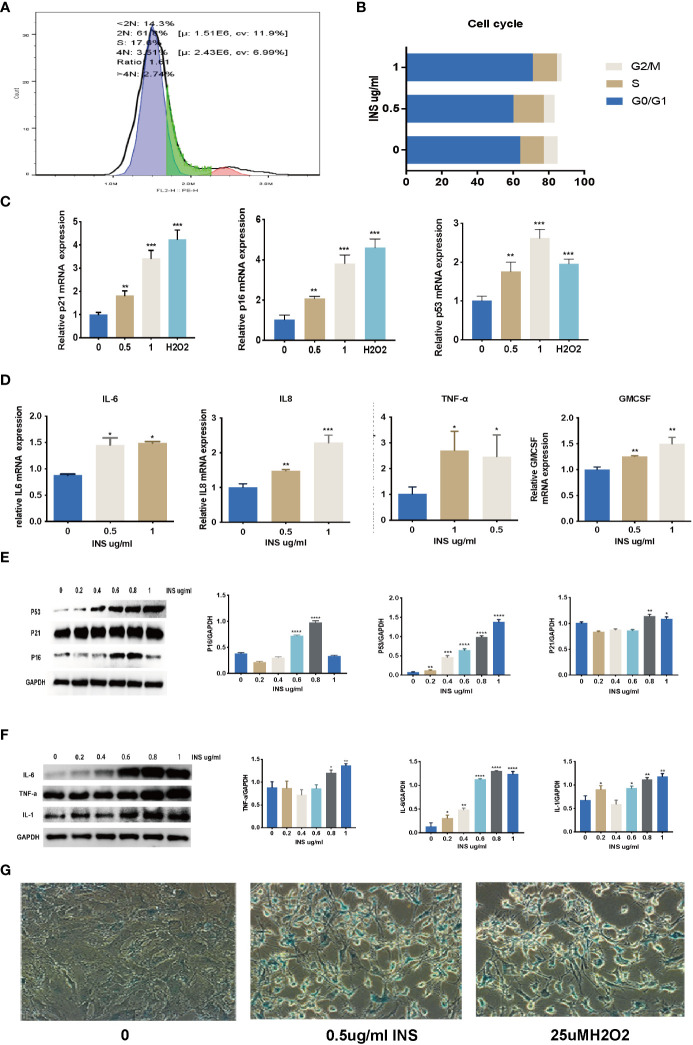
Long time exposure to high dose insulin induced granulosa cell senescence. KGN cells were treated with solvent, 0.5, and 1 µM insulin, respectively. **(A, B)** Cell cycles were detected by flow cytometry after treatment with 0.5 uM or 1 uM insulin for 72 h. **(C, D)** RNA was extracted from KGN cells. Relative mRNA expression of *p21, p53, p16, IL6, IL8, TNF-a*, and *GM-CS*F were examined by qPCR. n=3; t-test. **(E, F)** RNA was extracted from the cells. *p21, p53, p16, IL1, IL6*, and *TNF-a* protein expression were examined by western blot analysis. n=3; t-test. n=3 with one repeat in each assay; one-way ANOVA. **(G)** KGN cells with strong SA-β-Gal expression were detected induced by the high dose insulin. *: P<0.05, **: P<0.01, ***: P<0.001, ****: P<0.0001 compared to group “0”.

As shown in **Figures 3A, B**, after a 72-h manipulation of 0.5 µg/ml and 1 ug/ml insulin, the percentage of G0/G1 phase cells increased significantly in the 1 ug/ml insulin-treated group compared to the control group. The mRNA expression of *p21*, *p16*, and *p53* increased significantly in the 0.5– 1 ug/ml insulin-treated groups and H_2_O_2_-treated group compared to the control group (**Figure 3C**). Moreover, cytokines of SASP such as IL6, IL8, TNF-a, and GM-CSF mRNA expression were significantly increased in the 0.5 ug/ml and 1 ug/ml insulin groups (**Figure 3D**). As shown in **Figures 3E, F**, the protein expression of *p21, p16, p53, IL1, IL6*, and *TNF-a* significantly increased in the high dose insulin groups (0.6–1ug/ml) compared to the control group after 72 h. To further confirm our hypothesis, KGN cells treated for 72 d with 0.5 ug/ml and 1 ug/ml insulin were then fixed and subjected to senescence associated β-galactosidase (SA-β-Gal) staining. Strikingly, more than 90% of the KGN cells treated with high dose insulin were positive for SA-β-Gal staining (**Figure 3G**).

### 3.4 Higher levels of biomarkers of aging in HFD-fed mice

**Figure 4:**
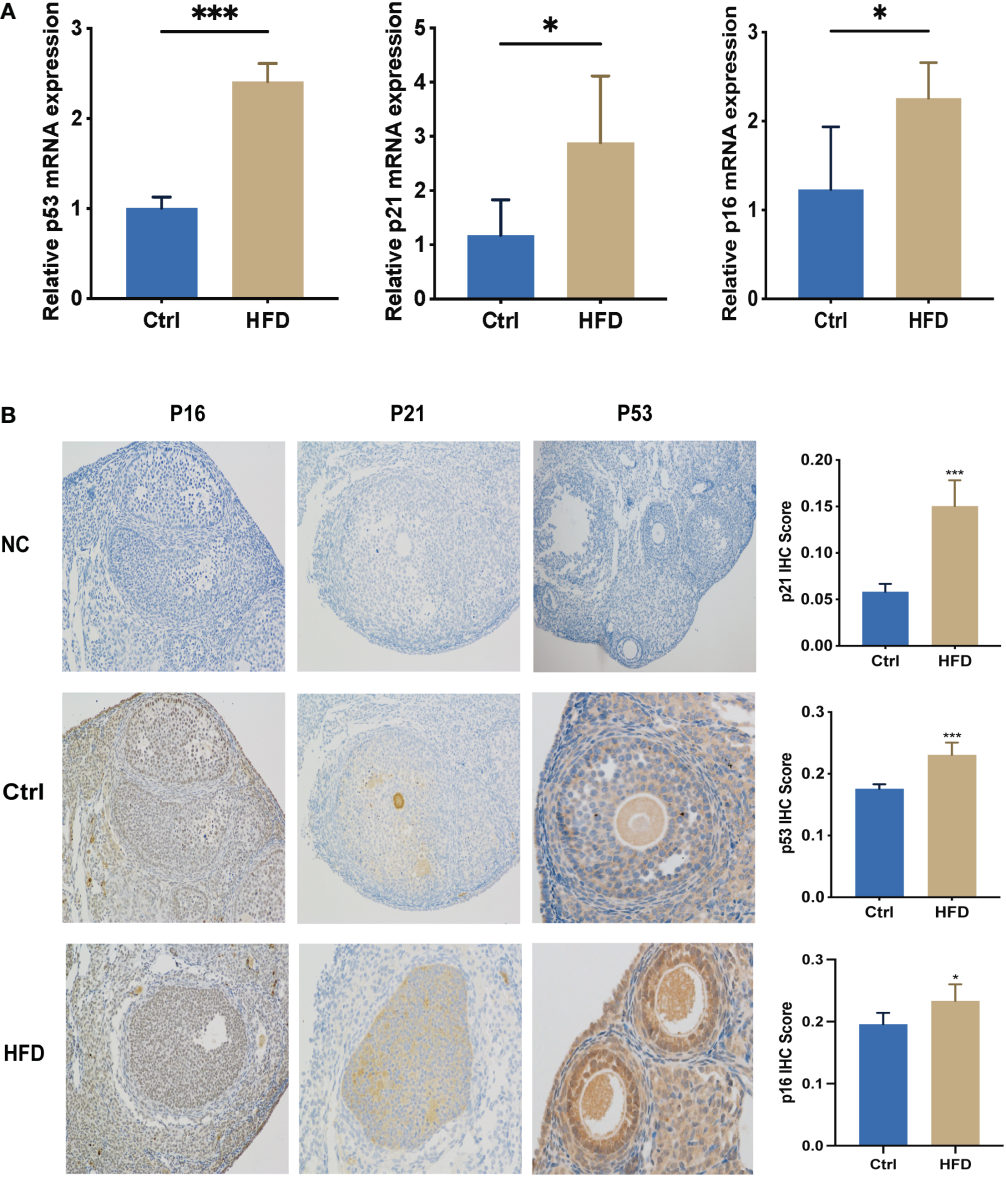
Levels of senescence-associated biomarkers in HFD-fed mice were greatly increased. **(A)** RNA was extracted from the ovaries of mice in Ctrl and HFD groups, n=3 at each time point and each group; unpaired t-test. **(B)** IHC of *p16, p53, and p21* in Ctrl (n = 5) and HFD (n = 5) mouse ovaries. The error bars indicate the mean values ± SDs, unpaired t-test. *: P<0.05, ***: P<0.001 compared to “Ctrl”.

Female C57B6/L mice were supplemented with a normal diet (Ctrl) or high-fat-diet (HFD) for 9 weeks. Relative mRNA and protein expression of *p53*, *p21*, and *p16* were detected by RT-PCR and IHC. As shown in **Figures 4A, B**, the level of *p53*, *p21*, and *p16* mRNA and protein expression in HFD were significantly increased compared to that of Ctrl.

### 3.5 RNA-seq expression analysis for the high dose insulin-treated KGN cells

RNA-seq gene expression analysis in the KGN cells treated with PBS (control group) and 1 ug/ml insulin (insulin group) was assessed. According to the results in **Figure 3**, 1 ug/ml had a more pronounced effect in causing KGN cell senescence; therefore, we selected 1 ug/ml insulin rather than 0.5 ug/ml insulin to treat KGN cells for RNA-seq expression analysis. The raw data of RNA-seq gene expression analysis can be found in https://www.ncbi.nlm.nih.gov/geo/query/acc.cgi?acc=GSE223248.

**Figure 5:**
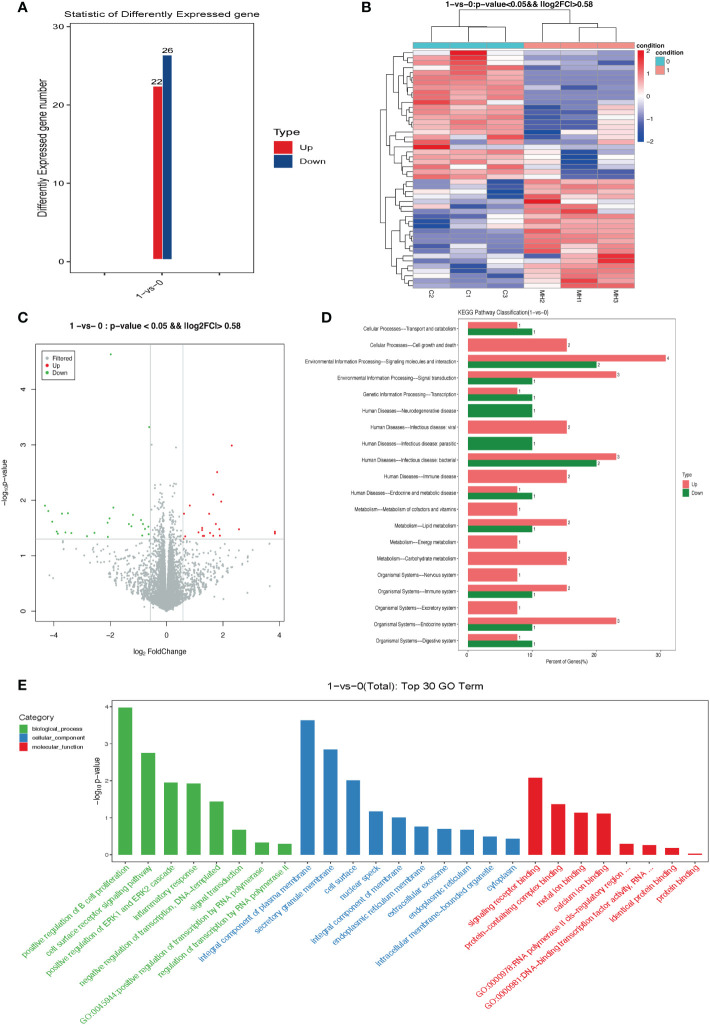
Differentially expressed genes (DEGs) in KGN cells treated with high dose insulin. **(A)** Column graph of DEGs in different treatment groups showing both up-regulated and down-regulated genes. **(B)** Heatmap. Green indicates repressed mRNA levels and red indicates elevated levels after insulin treatment. (log2 fold ratio ≥ 0.58; P < 0.01). **(C)** The volcano plot of DEGs present, in which green dots indicate down-regulated genes and red dots indicate up-regulated genes in response to insulin treatment. **(D)** KEGG enrichment analysis of differentially expressed genes. **(E)** GO terms of the top 30 enriched genes. The GO enrichment analysis grouped these differently expressed genes into functional groups. The green column represents biological processes, the blue column represents cellular components, and the red column represents molecular components. GO, Gene Ontology.

In comparison with the control group, 22 DEGs were up-regulated and 26 DEGs were down-regulated in the insulin group (**Figure 5A**). A heat map (**Figure 5B**) and volcano plot (**Figure 5C**) were plotted using the fold change and corrected P-values. As shown in **Figure 5D**, KEGG pathway analysis was then performed on the DEGs, and a total of 47 pathways were found to be involved; they were mainly enriched for cell growth and death, signaling molecules and interaction, and endocrine system. (**Figure 5D**). Top 30 gene ontology (GO) terms demonstrated that, after a 72-h 1 ug/ml insulin treatment the DEGs were mainly enriched to the ERK1 and ERK2 cascade pathway, integral component of plasma membrane, and signaling receptor binding. (**Figure 5E**).

### 3.6 RT-PCR was performed to verify the RNA-seq results

**Figure 6:**
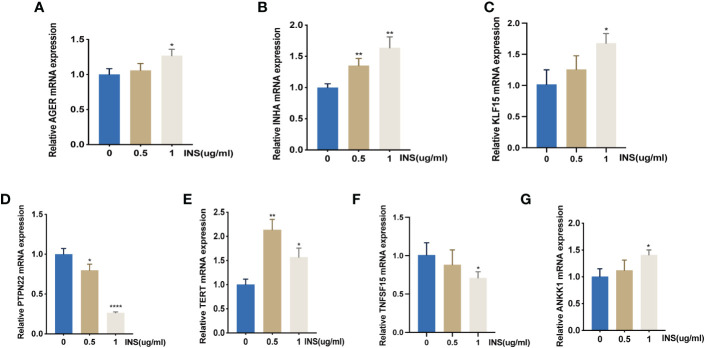
qRT-PCR verification of RNA-Seq analysis of gene expression. *: **(A–G)** The mRNA expression of AGER, INHA, KLF15, PTPN22, TERT, TNFSF15, and ANKK1 were detected by *: P<0.05, **: P<0.01, ****: P<0.0001 compared to group “0”.

To validate the RNA-Seq results, six genes were chosen for qRT-PCR analysis. Glycosylation end-product-specific receptor (AGER), Enoyl acyl carrier protein reductase (INHA), Kruppel-like factor 15 (KLF15), Telomerase Reverse Transcriptase (TERT), and Ankyrin repeat and kinase domain containing 1 (ANKK1) were up-regulated in KGN cells treated with 0.5 ug/ml and 1 ug/ml insulin. Additionally, protein tyrosine phosphatase non-receptor 22 (PTPN22) and tumor necrosis factor ligand superfamily member 15 (TNFSF15) were significantly down-regulated after insulin treatment. The qRT-PCR results were in accordance with the RNA-Seq results, indicating the data reliability of RNA-seq (**Figure 6**).

### 3.7 Gengnianchun attenuated insulin resistance and mitigated damage to the ovarian reserve

**Figure 7:**
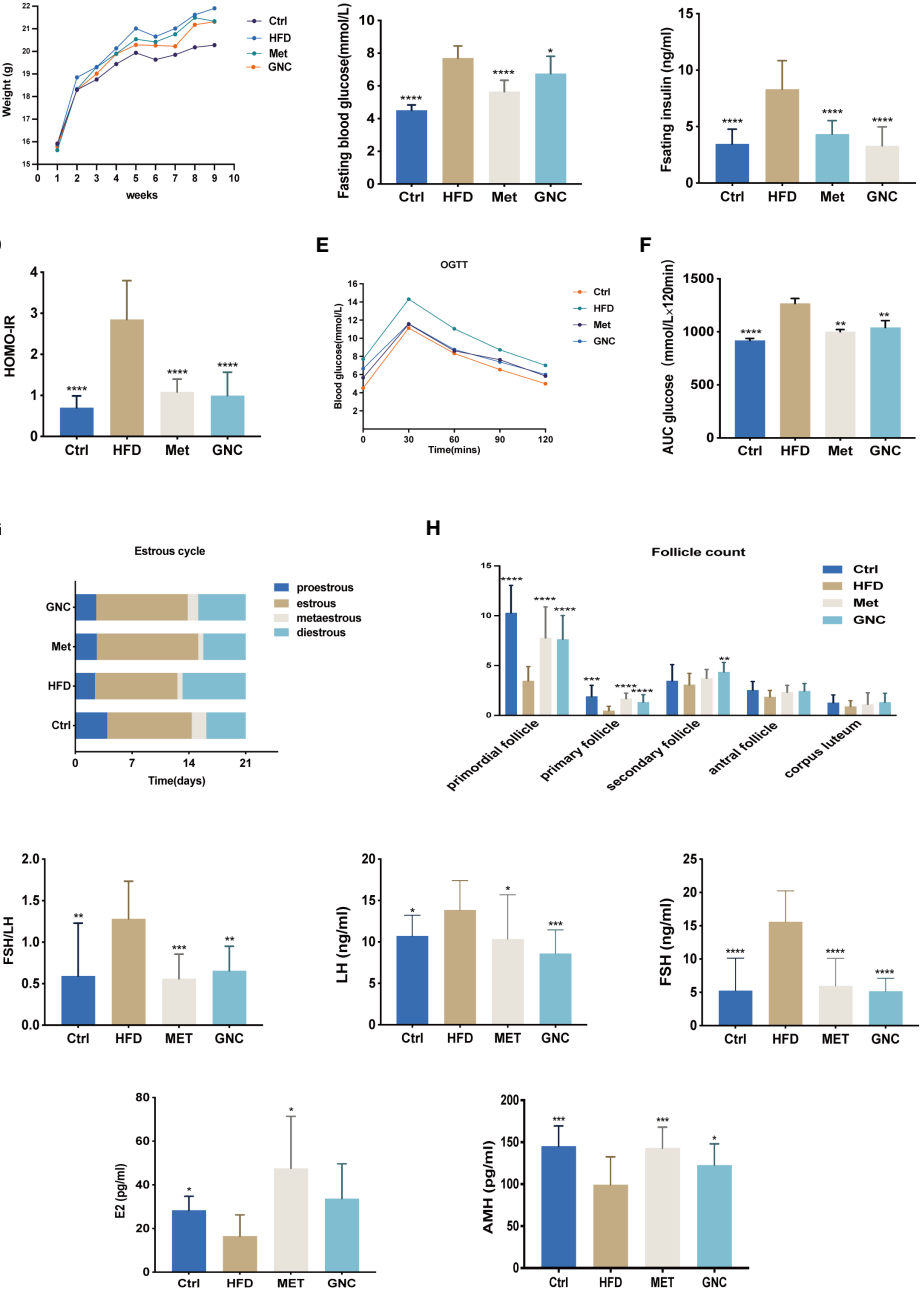
Gengnianchun reversed both HFD induced-insulin resistance and decreased ovarian function. **(A)** Weight of mice in the control group (Ctrl), HFD-fed group (HFD), Metformin group (Met), and Gengnianchun-fed group (GNC). **(B–F)** Fasting blood glucose, HOMA-IR, OGTT, and area under the curve (AUC) of mice in each group were assessed after a 12–14-h fast. **(E–G)** Different stages of the estrus cycle were assessed by collecting vaginal smears at same time point every day. Estrus cycle was assessed every day for the last 3 weeks, n=14 with one repeat in each assay. **(H)** Detailed counting of follicles at different stages, n=14 with one repeat in each assay; t-test. **(I)** Serum FSH, LH, E2, AMH, and fasting insulin were determined by ELISA with a 12–14-h fast before sacrifice; FSH/LH was calculated, n=14 with one repeat in each assay; *: P<0.05, **: P<0.01, ***: P<0.001, ****: P<0.0001 compared to group “Ctrl”.

Female C57B6/L mice were fed with a saline solution (Ctrl), HFD (HFD), 200 mg/kg·d Metformin (Met), or Gengnianchun (GNC). As **Figure 7A** shows, the body weight of HFD was much higher than for Ctrl, Met, or GNC. Basal glucose, insulin concentrations, and HOMA-IR were significantly increased in HFD compared with the other groups following 12–14 h of fasting (**Figures 7B–D**). We also found that an HFD resulted in a higher level of significance (P = 0.0001) in area under the curve (AUC) of OGTT compared with Ctrl. Moreover, Met and GNC were able to reverse this adverse change (**Figures 7E, F**). As shown in **Figure 7G**, compared with Ctrl, the percent of diestrus mice in HFD was much higher, while the increase was reversed in Met and GNC. Moreover, the numbers of primordial follicles (P < 0.001), primary follicles (P < 0.01), and secondary follicles (P < 0.05) in HFD were significantly lower than those of Ctrl, which were also reversed in Met and GNC (**Figure 7H**). **Figure 7I** shows that the levels of FSH and the ratio of FSH/LH in HFD were significantly higher than those mice in Ctrl,Met, and GNC groups, while the concentrations of AMH and E2 in HFD were significantly lower than those mice in Ctrl,Met, and GNC groups These findings all provide evidence for the therapeutic efficacy of the Gengnianchun formula on ovarian function.

### 3.8 Gengnianchun reduced aging-related mRNA and protein levels

**Figure 8:**
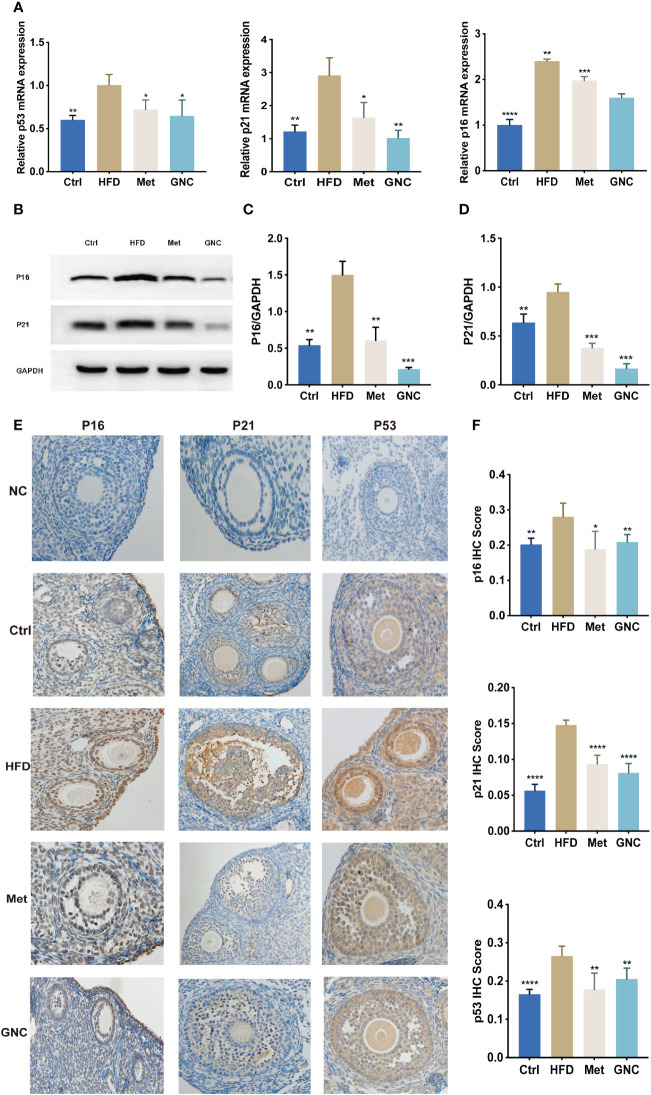
Gengnianchun reduced aging-related mRNA and protein levels. **(A)** The relative mRNA expression of *P53, P16*, and *P21* in ovaries of mice in each group were detected by qPCR. n=3; t-test. **(B–D)** The protein expression of *P53, P16*, and *P21* in the ovaries of mice in each group. n=3; t-test. **(E, F)** The protein expression of *p16, p53*, and *p21* in Ctrl (n = 5) and HFD (n = 5) mouse ovaries were determined by IHC. n=5; *: P<0.05, **: P<0.01, ***: P<0.001, ****: P<0.0001.

As shown in **Figure 8A**, gene expressions of *P53, P16*, and *P21* were significantly upregulated in HDF compared to Ctrl, and the use of metformin and GNC formula reversed the up-regulation of these proteins. The levels of protein expression of *P53, P16*, and *P21* were tested using western blotting (WB) and IHC (**Figures 8B–F**) and the results showed that the relative levels of proteins in HFD were significantly higher than the Met and GNC groups, thus confirming the efficacy of metformin and GNC formula *in vivo*.

## 4 Discussion

DOR has a significant impact on female reproductive health and pregnancy rates. It is common for genetic factors, environmental pollution, and infections to contribute to DOR in the modern world. It is well known that insulin resistance and hyperinsulinemia always exist simultaneously (28), and these are associated with PCOS. However, during routine clinical practice, we found that women with IR were also more likely to suffer from DOR, and our experience showed that the GNC formula could be effective in attenuating diminished ovarian function.

In this study, we first investigated the effects of IR on ovarian reserve in mice. There is now widespread agreement that HFD feeding results in IR in C57BL/6 mice (29, 30). Consistent with these previous findings, we established an IR mouse model by exposing mice to an HFD for 9 weeks, which resulted in increased weight, increased levels of fasting blood glucose and fasting insulin, and increased the HOMA-IR index in HFD mice compared with Ctrl mice. Furthermore, we found that HFD had lower counts in the numbers of primordial follicles, primary follicles and secondary follicles, longer diestrus, and higher levels of sex hormones compared with Ctrl, which supported the IR-diminished ovarian reserve of mice.

To further explore the specific mechanism of IR on the ovarian reserve *in vivo*, we treated the cells with high concentrations of insulin. We found that KGN cells underwent cell senescenceafter treatment with high concentrations of insulin. SASPs, which are a prominent source of chronic inflammation in the aging microenvironment (31, 32), are associated with the increased activation of nuclear factor kappa-B (NF-kB, also known as NF-kappaB) pathway (33). Based on the results of RNA-seq of insulin-treated KGN cells, we found that AGER, also known as RAGE, was up-regulated. AGER was found to be able to activate the NF-kB signaling pathway and ERK signaling pathway (34, 35), which may indicate a potential molecular mechanism of high concentrations of insulin leading to cell senescence.

To further verify the adverse effect of IR on ovarian reserve *in vitro*, metformin was used to alleviate HFD-induced IR. Moreover, a comparison of ovarian function was made between the HFD and Met groups. Metformin is the drug most commonly used to alleviate IR (36), with a dose of 200 mg/kg·d based on previous study (37). Our results showed that metformin decreased HFD-induced IR Notably, we also found that a reversal of ovarian reserve existed in Met mice compared with HFD, which supported previous findings that IR results in DOR.

According to TCM theory, GNC has a kidney/liver tonifying effect that is used to alleviate declining functions related to aging. GNC has been shown to improve learning and memory, delay skin aging, and enhance resistance to oxidative stress (17, 18). Furthermore, GNC can significantly extend lifespan and mitigate damage to the ovarian reserve according to our previous study (19, 20).

According to our results, GNC formula significantly reduced IR and increased the ovarian reserve. The expression levels of *p53, p21*, and *p16* detected by WB in GNC were much lower than those in Met, which suggested that GNC was more effective in attenuating ovarian aging compared to metformin. However, the protein expression levels of *p16, p53*, and *p21* detected by IHC showed no notable differences among the groups. Considering the higher accuracy of WB quantification over IHC, we conclude that GNC was more effective in attenuating ovarian aging than metformin. This suggests that other potential therapeutic targets of GNC may exist, and these require further exploration.

According to our results, metformin and GNC were able to alleviate the IR-induced diminished ovarian reserve, which is consistent with research showing that metformin can alleviate aging-related diseases and improve health span (38, 39). Furthermore, the SASP of senescent cells and accumulation of senescent cells are the major causes of excessive inflammation in age-related disorders (40), which is consistent with the elevated inflammatory factors in DOR (41, 42). However, we must acknowledge that there were limitations in the present study. It is commonly known that oocyte-granulosa cell communication plays a crucial role in the development of follicles (43). Thus, one limitation of our study was that the effect of the senescent granulosa cells on oocytes was not described.

In clinical practice, we found that patients with IR were more vulnerable to DOR, which has not been previously reported. Despite its preliminary character, this study clearly indicated the adverse effects of IR on ovarian reserve and granulosa cells, none of which have been previously reported. One important future direction of our study is to probe the specific mechanisms of IR on senescent granulosa cells; next, we want to understand whether these cells have adverse effects on oocytes. We also assume that the SASP of senescent cells may play a key role in oocyte dysfunction. Moreover, we found that the P53 gene was up-regulated in mouse ovaries based on IHC images. This poses an additional hypothesis regarding the involvement of IR and senescent granulosa cells in oocyte aging that warrants further investigation.

In conclusion, we identified that IR decreased ovarian reserve *via* modulating granulosa cell senescence and GNC had preservatory effects upon the ovarian reserve through regulating insulin resistance. Remarkably, these results from a mouse model and KGN cells appear to agree with our previous clinical observations. However, the detailed mechanisms of hyperinsulinemia and senescent oocyte granulosa cells need to be further investigated.

## Data availability statement

The data presented in this study has been deposited and made publicly available in an acceptable repository. The raw data of RNA-seq gene expression analysis can be found in [https://www.ncbi.nlm.nih.gov/geo/query/acc.cgi?acc=GSE223248].

## Ethics statement

The animal study was reviewed and approved by the Animal Experimental Ethical Committee of Fudan University.

## Author contributions

WW and HG contributed to the conception and design of the study. LG and HG performed the majority of experiments, data acquisitions, analyzed data, and wrote the manuscript. YR assisted with animal experiments. LQ helped analyze results. WW and ML supervised the study and helped to finalize the manuscript. All authors contributed to the article and approved the submitted version.
